# Dual-Donor-Induced Crystallinity Modulation Enables 19.23% Efficiency Organic Solar Cells

**DOI:** 10.1007/s40820-024-01576-1

**Published:** 2024-11-27

**Authors:** Anhai Liang, Yuqing Sun, Sein Chung, Jiyeong Shin, Kangbo Sun, Chaofeng Zhu, Jingjing Zhao, Zhenmin Zhao, Yufei Zhong, Guangye Zhang, Kilwon Cho, Zhipeng Kan

**Affiliations:** 1 Center On Nanoenergy Research, Institute of Science and Technology for Carbon Peak & Neutrality, School of Physical Science & Technology, Guangxi University, Nanning, 530004 People’s Republic of China; 2 Department of Chemical Engineering, Pohang University of Science and Technology, Pohang, 37673 South Korea; 3 College of New Materials and New Energies, Shenzhen Technology University, Shenzhen, 518118 People’s Republic of China; 4 Zhejiang Engineering Research Center for Fabrication and Application of Advanced Photovoltaic Materials, School of Materials Science and Engineering, NingboTech University, Ningbo, 315100 People’s Republic of China; 5 State Key Laboratory of Featured Metal Materials and Life-Cycle Safety for Composite Structures, Nanning, 530004 People’s Republic of China

**Keywords:** Trap-assisted charge recombination, Photoluminescence, Miscibility, Current leakage, Power conversion efficiency

## Abstract

Trap-assisted charge recombination is one of the primary limitations of restricting the performance of organic solar cells. However, effectively reducing the presence of traps in the photoactive layer remains challenging. Herein, wide bandgap polymer donor PTzBI-dF is demonstrated as an effective modulator for enhancing the crystallinity of the bulk heterojunction active layers composed of D18 derivatives blended with Y6, leading to dense and ordered molecular packings, and thus, improves photoluminescence quenching properties. As a result, the photovoltaic devices exhibit reduced trap-assisted charge recombination losses, achieving an optimized power conversion efficiency of over 19%. Besides the efficiency enhancement, the devices comprised of PTzBI-dF as a third component simultaneously attain decreased current leakage, improved charge carrier mobilities, and suppressed bimolecular charge recombination, leading to reduced energy losses. The advanced crystalline structures induced by PTzBI-dF and its characteristics, such as well-aligned energy level, and complementary absorption spectra, are ascribed to the promising performance improvements. Our findings suggest that donor phase engineering is a feasible approach to tuning the molecular packings in the active layer, providing guidelines for designing effective morphology modulators for high-performance organic solar cells.

**Supplementary Information:**

The online version contains supplementary material available at 10.1007/s40820-024-01576-1.

## Article Highlights

- By modulating the crystalline properties of the active layer with dual donors, the efficiency of organic solar cells reaches 19.23%.
- The introduction of PTzBI-dF suppresses the accumulation of traps and charge recombination, allowing ternary devices to maintain 82% of their initial power conversion efficiency (PCE) after illumination for 800 h.
- The dual-donor strategy for modulating the crystallinity of the active layer is applicable to a variety of Y6 derivatives, and the increase in PCE exceeds 1%.

**Supplementary Information:**

The online version contains supplementary material available at 10.1007/s40820-024-01576-1.

## Graphical abstract

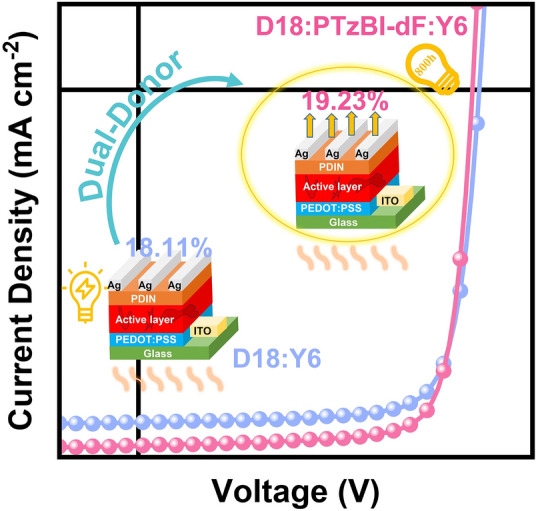

## Introduction

Organic solar cells (OSCs) attract broad research interests due to their properties, such as portability, flexibility, and printability [1–9]. Owing to the development of nonfullerene acceptors (NFAs), for instance, Y6 and its derivatives, the power conversion efficiency (PCE) of both single-junction OSCs and tandem devices has exceeded 20% [10, 11]. Besides the chemical structures of the donor or acceptor materials, the nongeminate recombination losses, especially trap-assisted charge recombination, are the primary limiting factors to improving the PCE of OSCs further. Therefore, reducing nongeminate recombination is a practical approach to enhance the performance of OSCs. To this end, optimizing methods, such as the selection of solvents, the regulation of additives, the introduction of a third component, interface engineering, and various post-treatment methods, were applied [12–20].

Adding a third component is demonstrated as one feasible way to broaden the absorption spectra, regulate the energy level, improve the crystallinity/phase separation, and increase the charge transport and transfer properties, potentially inhibiting the free charge recombination for enhancing the photovoltaic performance of OSCs [21–28]. A small NFA, m-BTP-PhC6, was used to regulate the active layer morphology as a second acceptor. Due to the good compatibility between m-BTP-PhC6 and D18-Cl: Y6 blend and the matching quasi cascade energy level arrangement, the exciton separation efficiency was improved, and the trap-assisted charge recombination loss was effectively reduced, leading to improved devices’ performance [29]. Besides introducing NFAs as the third component, dual donors with good miscibility were usually used for efficient ternary OSCs [30–32]. A polymer donor S3 with 20% chlorinated thiophene units was synthesized and added to PM6:Y6 as the third component. The dual donors formed an alloy-like thin film due to their excellent compatibility, resulting in enhanced charge generation and extraction, and finally achieved a PCE of 17.53% [33]. When D18-Cl was added to PM6:L8-BO, an alloy morphology was formed due to the close chemical structure of D18-Cl and PM6. Additionally, with the variation of D18-Cl content, the energy levels, including the charge transfer state energy (E_CT_), were tuned. As a result, the energy between the optical band gap and the E_CT_ was narrowed, leading to reduced energy loss and, thus, a PCE of 19.22% [34]. With considerable efforts in developing the additional component, adopting the ternary active layer becomes a common strategy to improve the performance of OSCs, and the screening of miscibility between the guest and host materials is proved as one effective method to select the third component. However, the challenge of choosing the third component that can optimize the morphology of the active layer remains. We speculate that materials with structural similarity but different aggregation features could be a direct material selection approach. Therefore, it is worth exploring the impact of material crystallinity on the donor–acceptor phase segregation, the trap-assisted charge recombination, and the influence on the photovoltaic performance.

In this work, we systematically investigated the effect of dual-donor-induced active layer phase regulation on trap-assisted charge recombination and, thus, the performance of OSCs. The active layer composed of D18-Cl and Y6 was chosen as the model system. A wide band gap polymer donor, PTzBI-dF, showing lower crystallinity in thin films relative to D18-Cl, was selected as an additional component for fine-tuning the morphology of the D18-Cl: Y6 active layer. The introduction of PTzBI-dF into the host blend (D18-Cl: Y6) leads to dense and ordered molecular packing, improving the crystallinity of the active layer and providing pathways for efficient exciton dissociation and charge transport. Consequently, the OSCs composed of D18-Cl: PTzBI-dF: Y6 attained a champion PCE of 18.60% with simultaneous improvement of open circuit voltage (*V*_OC_), short circuit current density (*J*_SC_), and fill factor (FF), outperforming that of the OSCs with D18-Cl: Y6 (17.90%). The improved efficiency was ascribed to decreased space charge accumulation, lower bimolecular recombination rate (1.23 × 10^–12^ *vs.* 0.91 × 10^–12^ cm^3^ s^−1^), and trap state density. The ternary device had a longer carrier lifetime (3.66 *vs.* 2.02 μs) and a faster charge extraction (0.26 *vs.* 0.47 μs), indicating that non-germinate recombination was effectively reduced. In addition, the optimized OSCs comprising D18: PTzBI-dF: Y6 achieved a PCE of 19.23%. The results provide guidance for modulating the crystalline properties of the active layer to reduce charge recombination and design efficient phase-optimizing materials.

## Experimental Section

### Materials

D18-Cl, Y6, and PDIN were purchased from Organtec Ltd. D18 and PTzBI-dF were obtained from Dongguan Volt-Amp Optoelectronics Technology Co., Ltd. PEDOT:PSS (CLEVIOSTM P VP AI 4083, Heraeus, Germany) was purchased from Xi‘an Yuri Solar Co., Ltd. All materials were used as received without further purification.

### Device Fabrication

Binary and ternary OSCs were fabricated with a conventional device configuration: ITO/PEDOT:PSS/active layers/PDIN/Ag. All devices were measured under AM 1.5G (100 mW cm^−2^) illumination. The glass substrate was coated with indium tin oxide (ITO, 15 Ω sq^−1^) with a device area of 0.04 cm^2^. The substrate was cleaned sequentially with dishwashing liquid, deionized water, acetone (15 min), deionized water, and isopropyl alcohol (15 min). The ITO glasses were treated with UV for 30 min in a UV-ozone chamber. A layer of PEDOT:PSS (~ 30 nm) (CLEVIOSTM P VP AI 4083, Heraeus, Germany) was spin-coated onto the UV-treated substrate, and the substrate was transferred to the glove box for active layer deposition.

All solutions were prepared in a nitrogen-filled glove box using chloroform (CF) as the solvent. The active layer solutions included D18-Cl:Y6, D18-Cl:PTzBI-dF:Y6 (mass ratio 1:1.6, 14.3 mg mL^−1^), and PTzBI-dF:Y6 (mass ratio 1:1.2, 16 mg mL^−1^). These solutions were heated for 2 h before use. The active layer solution was spin-coated at 4000 rpm for 20 s. Subsequently, the PDIN solution (2.0 mg mL^−1^ in methanol with 0.3 vol% acetic acid) was spin-coated on the active layer as the electron transport layer. The substrate was then pumped into a high vacuum at a pressure of 2 × 10^−7^ torr, where a 100 nm thick Ag layer was thermally evaporated onto the active layer.

## Results and Discussion

### Photophysical Properties

**Fig. 1:**
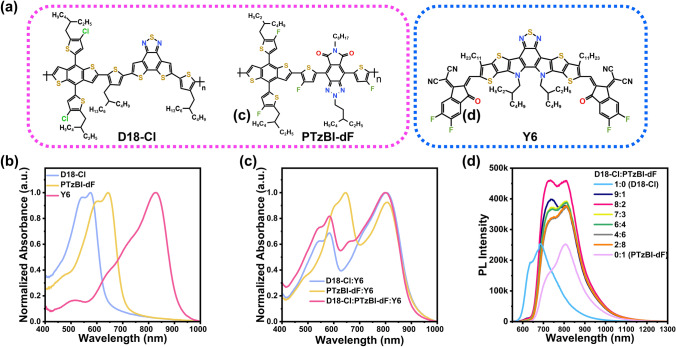
**a** Molecular structure of D18-Cl, PTzBI-dF, and Y6. **b** Normalized absorbance spectra of neat films D18-Cl, PTzBI-dF, Y6, and **c** blend films. **d** Photoluminescence spectra of D18-Cl: PTzBI-dF blend films with different proportions under 500 nm excitation

Figure 1a plots the chemical structure of D18-Cl, PTzBI-dF, and Y6. Figure 1b shows the absorption spectra of D18-Cl, PTzBI-dF, and Y6. The strong absorption peaks of D18-Cl, PTzBI-dF, and Y6 films are 575, 642, and 827 nm, respectively, and the light absorption of PTzBI-dF is complementary to absorptions of D18-Cl and Y6. As shown in Fig. 1c, after adding an appropriate amount of PTzBI-dF, the absorption intensity of the ternary blend film from 400 to 710 nm was enhanced, thus achieving enhanced photon capture capability, which is conducive to obtaining higher *J*_SC_ of the device. The optical band gap (E_opt_) was determined by the energy at the intersection of normalized absorption and photoluminescence (PL) spectra of neat films (see Fig. S1 and Table S1 for details). The highest occupied molecular orbitals (HOMO) of D18-Cl, PTzBI-dF, and Y6 were determined by ultraviolet photoelectron spectroscopy (UPS), and the lowest unoccupied molecular orbitals (LUMO) was obtained by adding the E_opt_ to HOMO (Fig. S2). In detail, the LUMO of PTzBI-dF was − 3.75 eV, lower than that of D18-Cl (− 3.40 eV) and higher than that of Y6 (− 4.17 eV). The HOMO of PTzBI-dF was between D18-Cl (− 5.49 eV) and Y6 (− 5.63 eV). Thus, PTzBI-dF and the host system form an ideal cascade energy alignment that is favorable for charge separation, thereby improving charge transport and collection [35].

Figure 1d depicts the PL spectra of D18-Cl: PTzBI-dF blend films with various ratios, and the emission spectra of D18-Cl and PTzBI-dF are presented for comparison. The PL intensity of D18-Cl and PTzBI-dF is similar, with peaks at 685 and 806 nm, respectively, while the intensity of the blend films changes gradually with the ratio variation. When the ratio of D18-Cl: PTzBI-dF is 0.8:0.2, the PL intensity of the blend film is 1.8 times of the neat films D18-Cl or PTzBI-dF, indicating effective energy transfer from D18-Cl to PTzBI-dF [36, 37]. Besides, there is a large overlap between the PL spectra of the mixed donor and the absorption spectra of the acceptor (Fig. S3a). Generally, energy transfer occurs when one material’s (energy donor) emission spectrum overlaps with another material’s (energy acceptor) absorption spectrum [38, 39]. Therefore, energy transfer from D18-Cl: PTzBI-dF to Y6 is expected. When we excited the donor using a wavelength of 500 nm, the emission from 550 to 820 nm was quenched entirely. On the other hand, a peak at 934 nm is observed in the ternary blend because the energy transfer occurred between the donor and acceptor (Fig. S3b), followed by a hole transfer from Y6 to the donor. Additionally, D18-Cl: Y6 and PTzBI-dF: Y6 are effectively quenched, and the quenching efficiency reaches 89.1% and 91.8%. When 20% PTzBI-dF was added to the binary D18-Cl: Y6, the quenching efficiency of the ternary blend film was increased to 92.7%, indicating that PTzBI-dF promotes the exciton dissociation and the hole transfer in the ternary blend (Fig. S3c).

### Photovoltaic Performance

**Fig. 2:**
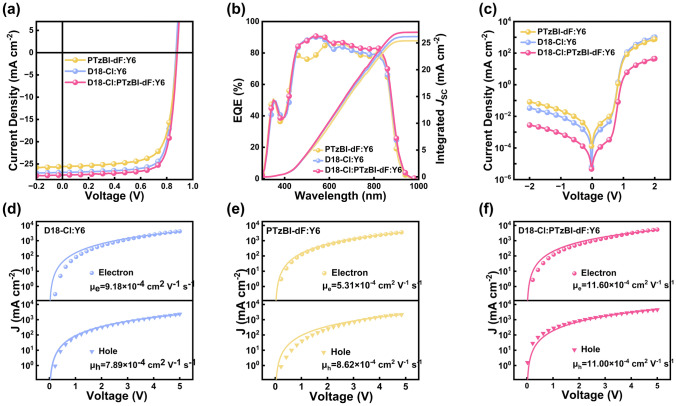
**a** *J-V* characteristics, **b** the external quantum efficiency (EQE) spectra, and **c** dark *J-V* curves of the optimized binary and ternary OSCs. **d**–**f** Dark current density as a function of the applied voltages. The solid lines are fit to the experimental data (scatter): **d** D18-Cl: Y6, **e** PTzBI-dF: Y6, and **f** D18-Cl: PTzBI-dF: Y6

To evaluate the impact of energy transfer between D18-Cl and PTzBI-dF on the performance of the devices, we fabricated OSCs composed of PTzBI-dF: Y6, D18-Cl: Y6, and D18-Cl: PTzBI-dF: Y6, respectively, with a conventional device structure of ITO/PEDOT: PSS/active layer/PDIN/Ag (Fig. S4). As presented in Fig. 2a and Table 1, the optimal PTzBI-dF: Y6-based device exhibited a *V*_OC_ of 0.879 V, *J*_SC_ of 25.63 mA cm^−2^, FF of 72.16%, and a PCE of 16.26%; and the optimal D18-Cl: Y6-based device exhibited a *V*_OC_ of 0.871 V, *J*_SC_ of 26.90 mA cm^−2^, FF of 76.37%, and a PCE of 17.90%. When we added different proportions of PTzBI-dF into the D18-Cl: Y6 system, the photovoltaic parameters changes were observed in the ternary devices, and the detailed information of the ternary OSCs was described in Table S2 and Section S2. Notably, adding 20% of PTzBI-dF to the active layer improved the *V*_OC_, *J*_SC_, and FF, leading to a PCE of 18.60%. The external quantum efficiency (EQE) spectra of the D18-Cl: Y6, PTzBI-dF: Y6, and D18-Cl: PTzBI-dF: Y6 devices were depicted in Fig. 2b. The ternary devices show an increased photo response in the wavelength range from *ca*. 515 to 800 nm, with a maximum value exceeding 90%, further confirming the contribution of PTzBI-dF. The integral current density values of PTzBI-dF: Y6, D18-Cl: Y6, and D18-Cl:PTzBI-dF:Y6 are 25.41, 26.19, and 27.00 mA cm^−2^, respectively. Compared with the *J*_SC_ measured under the illumination of AM 1.5G, 100 mW cm^−2^, the EQE integrated current *J*_cal, EQE_ is within 3% deviation (Table S3). Figure 2c shows the *J-V* measurement curve under dark conditions, and the ternary device exhibits the lowest dark current density, indicating suppressed current leakage and reduced space charge accumulation [40].

**Table 1:** Photovoltaic parameters of the optimum binary and ternary blend films under AM 1.5G and 100 mW cm^−2^ illumination

Photoactive layer	*V*_OC_ (mV)	*J*_SC_ (mA cm^−2^)	FF (%)	PCE (%)^a^
PTzBI-dF:Y6	879 (878 ± 2)	25.63 (25.24 ± 0.23)	72.19 (71.55 ± 0.33)	16.26 (15.86 ± 0.16)
D18-Cl:Y6	871 (874 ± 2)	26.90 (26.61 ± 0.09)	76.37 (76.23 ± 0.26)	17.90 (17.74 ± 0.07)
D18-Cl:PTzBI-dF:Y6	882 (880 ± 1)	27.50 (27.40 ± 0.12)	76.66 (76.55 ± 0.10)	18.60 (18.45 ± 0.06)
D18:Y6	867 (866 ± 2)	27.18 (26.76 ± 0.25)	76.80 (76.82 ± 0.32)	18.11 (17.80 ± 0.11)
D18:PTzBI-dF:Y6	869 (865 ± 2)	28.09 (27.50 ± 0.20)	77.15 (77.14 ± 0.36)	18.84 (18.55 ± 0.10)
D18:PTzBI-dF:Y6^b^	849 (846 ± 2)	29.08 (28.98 ± 0.17)	77.83 (77.50 ± 0.23)	19.23 (18.98 ± 0.17)

^a^Average values were obtained from 20 devices

^b^With 0.5% (by volume) 1,8-diiodooctane

To understand the accumulation of space charge in the investigated OSCs, we determined the hole mobility (*μ*_h_) and electron mobility (*μ*_e_) of the three blend films by using the space charge-limited current (SCLC) model. The related parameters and specific schematic diagram are summarized in Fig. 2d–f and Table S7. For the D18-Cl: Y6 system, *μ*_h_ and *μ*_e_ were measured at 9.18 × 10⁻^4^ and 7.89 × 10⁻^4^ cm^2^ V⁻^1^ s⁻^1^, respectively, yielding a *μ*_e_/*μ*_h_ ratio of 1.16. Conversely, in the PTzBI-dF: Y6 configuration, *μ*_e_ was recorded at 5.31 × 10⁻^4^ cm^2^ V⁻^1^ s⁻^1^ and *μ*_h_ at 8.62 × 10⁻^4^ cm^2^ V⁻^1^ s⁻^1^, inversing the mobility ratio to 0.62. Notably, an imbalance in *μ*_e_ and *μ*_h_, particularly when either is excessively low, can lead to free charge carrier recombination, thereby decreasing the FF [41]. Upon additional PTzBI-dF into the D18-Cl: Y6 system, an enhancement in both *μ*_e_ and *μ*_h_ was observed, increasing to 11.60 × 10⁻^4^ and 11.00 × 10⁻^4^ cm^2^ V⁻^1^ s⁻^1^, respectively. This adjustment resulted in a near-ideal *μ*_e_/*μ*_h_ ratio of 1.05, consistent with the exceptional FF and* J*_SC_ exhibited in these ternary OSCs. The balanced charge carrier mobility profile promotes efficient charge transport, facilitating higher FF [42].

### Charge Dynamics Characterizations

Next, we discussed the charge extraction and recombination of the investigated OSCs. *J-V* curves were recorded at various light intensities. By analyzing *J*_SC_ and *V*_OC_ as functions of light intensity, charge extraction and recombination were evaluated. Efficient charge extraction is indicated by the relationship of: $J_{\text{SC}} \propto {I}^{\alpha }$, where if α is close to 1, it indicates efficient charge extraction prior recombination [43]. Figure S7a shows the relationship between *J*_SC_ and light intensity, with all devices having α values close to 1, indicating efficient charge extraction. On the other hand, the ideality factor *n*, which reflects trap-assisted recombination, was determined using $V_{\text{OC}}\propto ln(I)nkT/q$, where n is the ideality factor, k is the Boltzmann constant, *T* is the Kelvin temperature, and *q* is the elementary charge [44]. An *n* value deviating from 1 indicates the presence of traps. As shown in Fig. S7b, the *n* values for D18-Cl:Y6, PTzBI-dF:Y6, and D18-Cl:PTzBI-dF:Y6 are 1.22, 1.49, and 1.04, respectively. The ternary OSC incorporating PTzBI-dF has the smallest n value, suggesting the least trap-assisted recombination.

**Fig. 3:**
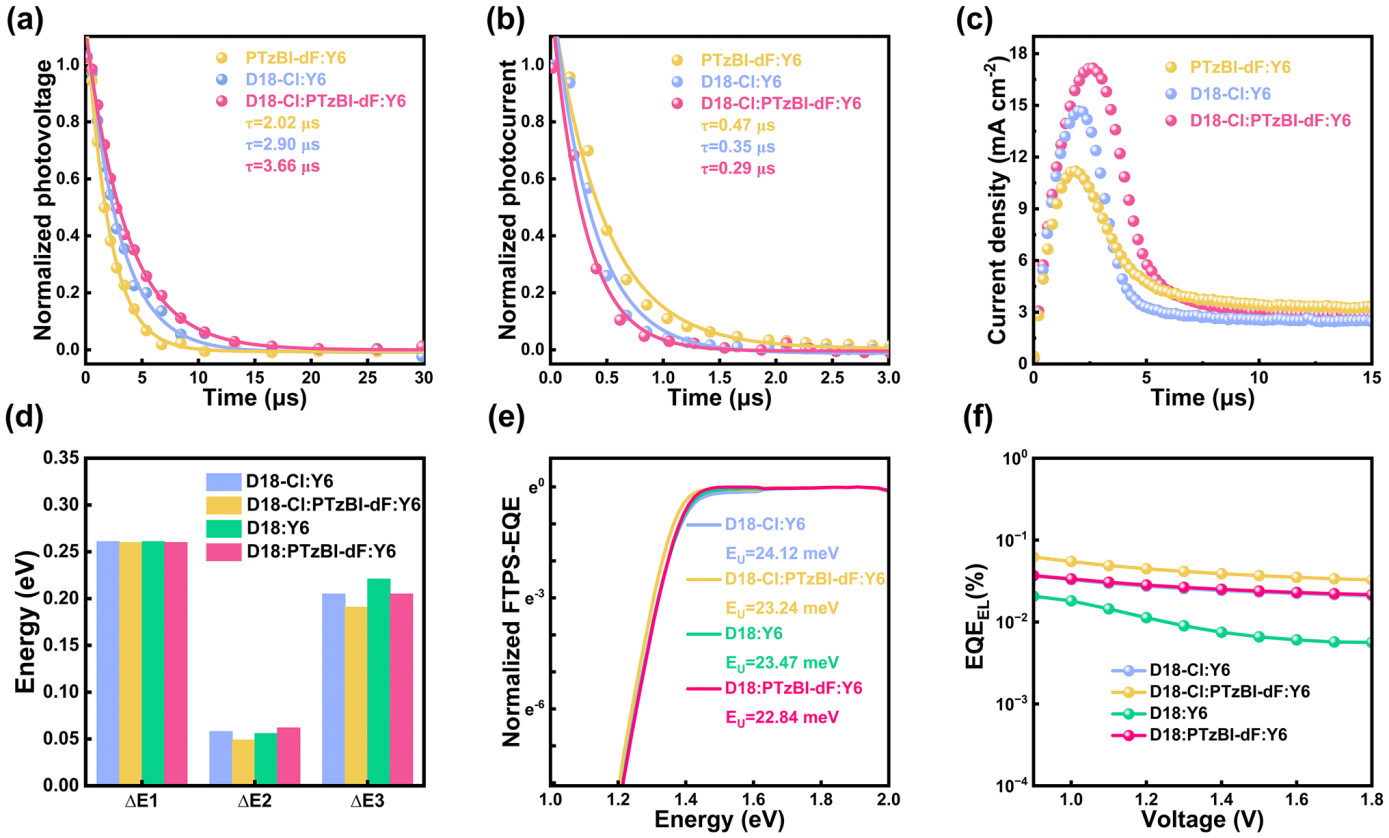
**a** Transient photovoltage, **b** Transient photocurrent, **c** Light-induced charge extraction by linearly increasing voltage current transient photo-induced charge extraction for optimal binary and ternary OSCs. **d** The comparison of detailed energy loss for binary and ternary OSCs. **e** E_U_ is Urbach energy, obtained from the FTPS-EQE curves with the exponential fitting ${\upalpha } \propto {\text{exp}}\left( {\frac{{E - E_{g} }}{{E_{U} }}} \right)$. **f** EQE_EL_ of binary and ternary devices

In addition to these qualitative results, we performed time-resolved characterizations on the devices to further investigate the charge dynamics. As plotted in Fig. 3a, transient photovoltage (TPV) was measured to derive the charge carrier lifetime of the devices [45, 46]. The decay time constants of PTzBI-dF: Y6, D18-Cl: Y6, and D18-Cl: PTzBI-dF: Y6 devices are 2.02, 2.90, and 3.66 μs, respectively. The enhanced charge carrier lifetime indicates that the introduction of the third component reduces nonradiative recombination, thus ensuring a longer time to extract charge. Then, the transient photocurrent (TPC) response of photovoltaic devices was measured, and the information of charge carrier extraction was obtained [47]. Figure 3b shows the carrier extraction time of the three devices. The charge extraction time of PTzBI-dF: Y6, D18-Cl: Y6, and D18-Cl: PTzBI-dF: Y6 devices are 0.47, 0.36, and 0.29 μs, respectively. The shortening of charge extraction time and the increase of carrier lifetime mean that the ternary device has higher charge mobility and less charge recombination, which was conducive to improving the *J*_SC_ and FF of the device. To quantify the charge recombination rate, we monitored the charge concentration as a function of extraction time by photo-induced charge carrier extraction by linearly increasing voltage (photo-CELIV) at various delay times in Fig. 3c.

The relationship between the charge carrier concentration and the bimolecular recombination rate of devices with different active layers is depicted in Fig. S9. A detailed description of the formulas for bimolecular recombination and corresponding fitting was provided in Section S7 and Table S8. Analysis reveals dispersion parameters (γ) for PTzBI-dF: Y6, D18-Cl: Y6, and D18-Cl: PTzBI-dF: Y6 systems as 0.89, 0.88, and 0.93, respectively. These values approach unity and signify less extent of traps within the device, while γ = 1 indicates a trap-free condition [48, 49]. The bimolecular recombination rates (β, measured at 10 ns) for these devices are quantified as 1.23 × 10⁻^12^ cm^3^ s⁻^1^ for PTzBI-dF: Y6, 1.17 × 10⁻^12^ cm^3^ s⁻^1^ for D18-Cl: Y6, and a notably lower 0.91 × 10⁻^12^ cm^3^ s⁻^1^ for the optimized ternary D18-Cl: PTzBI-dF: Y6 device. The reduction in the recombination rate and presence of traps, particularly in the ternary device, indicates a substantial suppression of nongeminate charge recombination processes, which aligns favorably with the observed enhancement in the performance of the ternary OSCs.

The suppression of nongeminate charge recombination was revealed by the energy losses analysis, which was determined by Fourier transform photocurrent spectroscopy external quantum efficiency (FTPS-EQE) and electroluminescence-EQE (EL-EQE) in Figs. 3d and S8. As shown in Fig. 3e, D18-Cl:PTzBI11-dF:Y6 and D18:PTzBI-dF:Y6 exhibit lower Urbach energy (E_U_) values than the binary devices, which are 23.24 and 22.84 meV respectively, and suggests that the introduction of PTzBI-dF can effectively reduce energy disorder and trap density by limiting charge recombination. As described in Fig. 3f and Table 2, the D18-Cl:Y6 and D18:Y6 devices exhibits weak EQE_EL_ of 3.6 × 10^–2^ and 2.0 × 10^–2^, respectively, with corresponding non-radiative loss values of 0.205 and 0.221 eV. In contrast, EQE_EL_ for D18-Cl:PTzBI-dF:Y6 and D18:PTzBI-dF:Y6 is 72% and 80% higher, with values of 6.2 × 10^–2^ and 3.6 × 10^–2^, resulting in non-radiative loss values as low as 0.191 and 0.20 eV. The lower E_U_ and low non-radiative loss values are in agreements with the reduced nongeminate charge recombination in the ternary OSCs.

**Table 2:** Detailed energy losses of organic photovoltaics (OPVs) based on binary and ternary devices

Active layer	*E*g (eV)	${V}_{OC}^{SQ}$(V)	*V*_OC_ (V)	${V}_{OC}^{rad}$(V)	*E*_loss_ (eV)	∆*E*_1_ (eV)	∆*E*_2_ (eV)	∆*E*_3_ (eV)	EQE_EL_ (%)
D18-Cl:Y6	1.400	1.140	0.871	1.081	0.524	0.261	0.058	0.205	3.6 × 10^–2^
D18-Cl:PTzBI-dF:Y6	1.379	1.119	0.882	1.070	0.500	0.260	0.049	0.191	6.2 × 10^–2^
D18:Y6	1.401	1.140	0.867	1.083	0.538	0.261	0.056	0.221	2.0 × 10^–2^
D18:PTzBI-dF:Y6	1.395	1.135	0.869	1.073	0.527	0.260	0.062	0.205	3.6 × 10^–2^

### Microstructure Characterizations

**Fig. 4:**
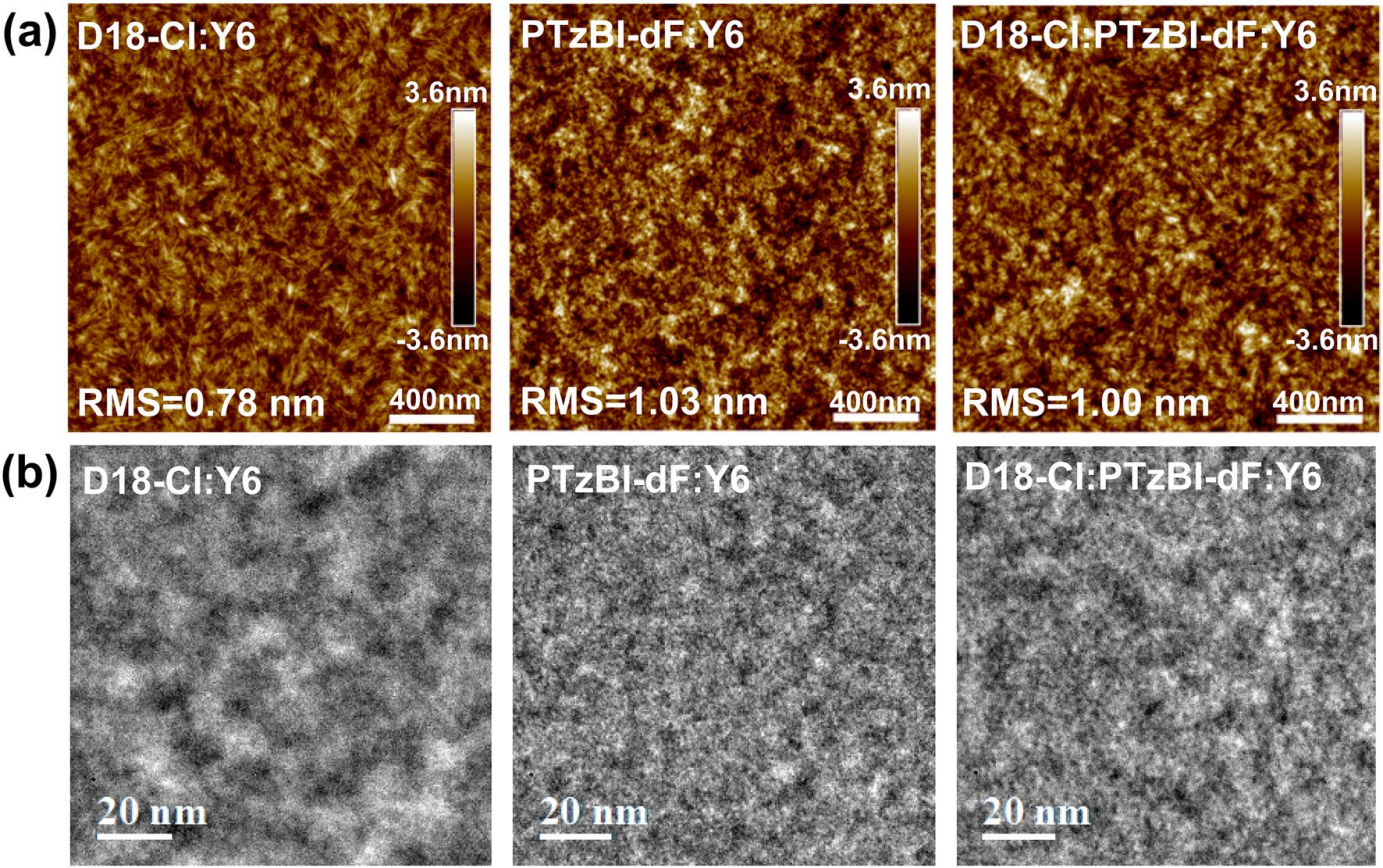
**a** Topography of PTzBI-dF: Y6, D18-Cl: Y6, and D18-Cl: PTzBI-dF: Y6. **b** Transmission electron microscopy images of PTzBI-dF: Y6, D18-Cl: Y6, and D18-Cl: PTzBI-dF: Y6

The electronic property changes usually originate from the thin film microstructure alternations. We conducted atomic force microscope (AFM) and transmission electron microscope (TEM) measurements to characterize morphological changes in the active layer. Figure 4a displays the topography of the thin films, and the results imply that the thin film of D18-Cl: PTzBI-dF: Y6 has better crystalline properties due to the slightly increased surface roughness. TEM images (Fig. 4b) demonstrate distinct aggregation characteristics with the various donor–acceptor (D-A) networks of different length scales. The PTzBI-dF: Y6 films exhibit less phase-separated D-A network and weaker crystallinity owing to the low crystalline nature of PTzBI-dF. In contrast, the D18-Cl: Y6 films exhibit a D-A network with larger scale phase separation and stronger crystallinity, which can adversely impact device performance due to the pronounced phase separation [52]. However, when 20% of D18-Cl is replaced with PTzBI-dF, as seen in the D18-Cl: PTzBI-dF: Y6 blend, the phase-separated D-A network and crystallinity are significantly optimized. The more homogeneous features in the TEM images potentially contribute to reduced trap-assisted charge recombination losses and enhance the PCE.

After gaining an in-depth understanding of the topography and nanostructure through AFM and TEM, we conducted contact angle tests to explore the surface energy and wetting coefficient (ω). As shown in Tables S9 and S11, the interfacial energy calculations revealed that when D18-Cl and PTzBI-dF are combined in a mass ratio of 0.8:0.2, the χ value for the D18-Cl:PTzBI-dF:Y6 ternary blend is 5.37 × 10^–2^ K, indicating better donor–acceptor miscibility compared to the binary film [53]. The wetting coefficient (ω) of PTzBI-dF, which determines its position in the ternary active layer, is given in the supporting information. The calculated value of ω_PTzBI-dF_ is 1.04, demonstrating that the incorporation of PTzBI-dF improves the miscibility between the donor and acceptor, thereby optimizing the donor–acceptor network.

**Fig. 5:**
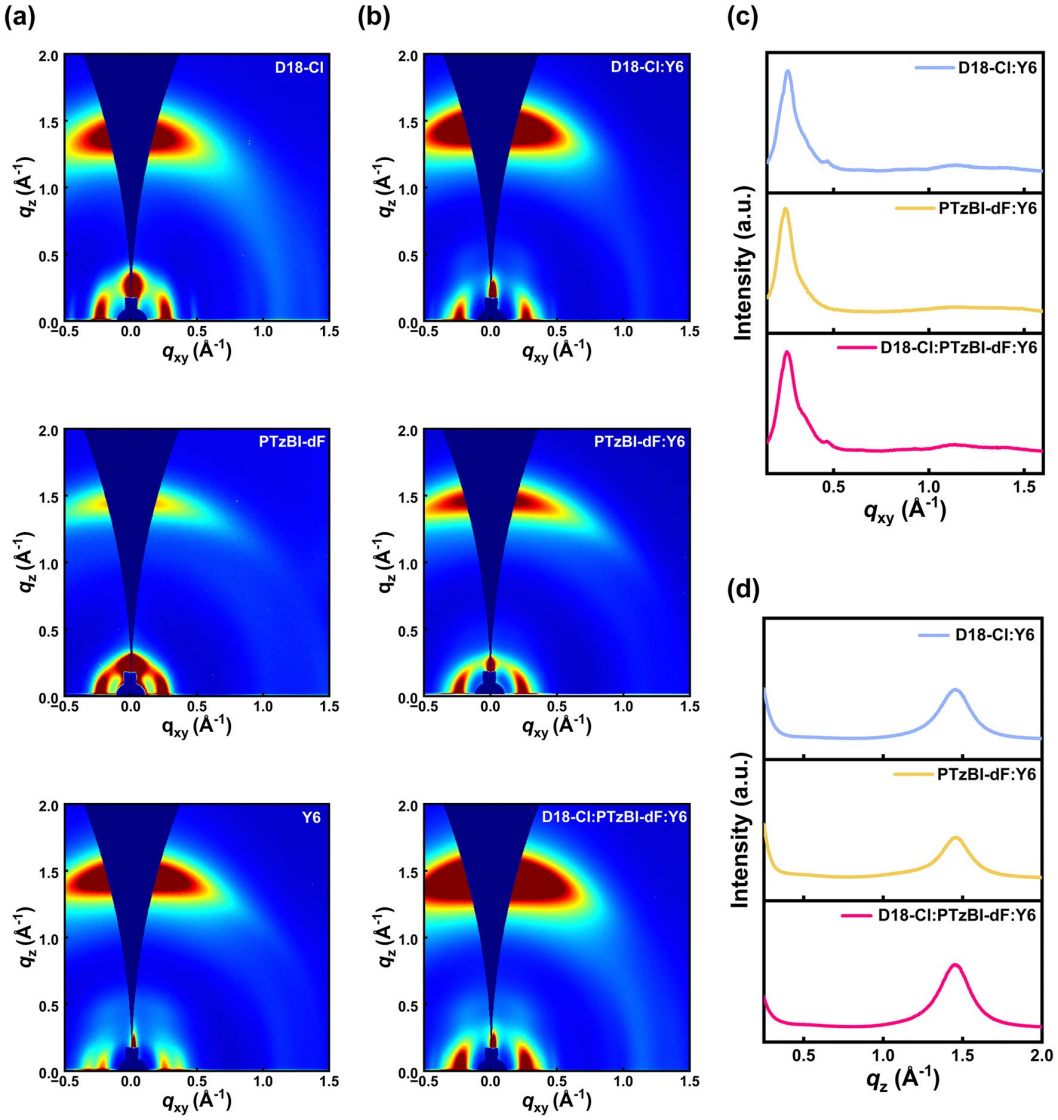
2D GIWAXS patterns and the line profiles of the in-plane (IP) and out-of-plane (OOP) directions of the neat and blend films. **a** 2D GIWAXS patterns of the neat D18-Cl, PTzBI-dF, and Y6. **b** 2D GIWAXS patterns of the neat D18-Cl: Y6, PTzBI-dF:Y6, and D18-Cl: PTzBI-dF: Y6. The corresponding **c** IP and **d** OOP line-cuts

To quantify the changes in the morphologies in the active layers, we performed grazing incidence wide-angle X-ray scattering (GIWAXS) to study the molecular arrangement and crystallinity of the films presented in Fig. 5a. The line cuts of the in-plane (IP) and out-of-plane (OOP) directions are in Fig. S11, and the peaks and crystal coherence length (CCL) of the neat D18-Cl, D18, PTzBI-dF, and Y6 in the IP and OOP direction are summarized in Tables S11 and S12. The D18-Cl, D18, and PTzBI-dF films exhibit distinct lamellar peaks at respective *q*_xy_ values of 0.258, 0.250, and 0.244 Å^−1^, indicative of their ordered molecular aggregation. In the OOP direction, there exist pronounced (010) π-π stacking peaks at *q*_z_ values of 1.392, 1.461, and 1.443 Å^−1^, respectively. The CCL value of PTzBI-dF is 28.99 Å, shorter than those observed for D18-Cl (30.90 Å) and D18 (31.42 Å). Analysis of the two-dimensional GIWAXS patterns for these neat films suggests that PTzBI-dF has a weaker π-π intermolecular interaction than D18-Cl and D18, confirming its lower crystalline features. Meanwhile, the neat Y6 film displays an IP (100) diffraction peak at *q*_xy_ = 0.238 Å^−1^ and an OOP (010) π-π packing peak at *q*_z_ = 1.452 Å^−1^.

Furthermore, the crystalline properties of the blend films were analyzed (Fig. 5b), with the IP and OOP profiles plotted in Fig. 5c, d. The key parameters, such as the CCL and π-π packing distance (d-spacing) of the thin films in the IP and OOP directions, are listed in Table S13 and Table S14. The peaks for D18-Cl and Y6 appear at 1.459 and 1.430 Å⁻^1^, respectively. The (010) diffraction peaks in the OOP direction have *d*-spacings of 4.31 Å for D18-Cl and 4.39 Å for Y6, with a CCL of 31.94 Å. For the PTzBI-dF: Y6 film, the peaks for PTzBI-dF and Y6 are at 1.461 and 1.404 Å⁻^1^, respectively. The (010) diffraction peaks have *d*-spacings of 4.30 Å for PTzBI-dF and 4.48 Å for Y6, with a CCL of 31.59 Å. The lower crystallinity of this active layer potentially leads to enhanced nonradiative recombination and unbalanced carrier migration [54, 55]. In the D18-Cl: PTzBI-dF: Y6 film, the donor and acceptor peaks are at 1.4578 and 1.430 Å⁻^1^, respectively. The (010) diffraction peaks in the OOP direction have *d*-spacings of 4.31 Å for the donor and 4.39 Å for the acceptor, with a CCL of 32.69 Å. These results indicate that fine-tuning the morphology with the polymer donor improves crystallinity and enhances molecular stacking order.

Motivated by the positive influence of PTzBI-dF on the morphology of the active layer and the device performance, we integrated it as a third component into the D18:Y6 blend. The molecular packing information was probed by GIWAXS, as shown in Figs. S12 and S13. The CCL changes from 34.06 Å (D18:Y6) to 34.27 Å (D18: PTzBI-dF: Y6), indicating slightly increased thin film crystallinity. Moreover, the incorporation led to a remarkable efficiency enhancement, with the D18: PTzBI-dF: Y6 ternary OSCs achieving an impressive 18.84% efficiency, ranking among the best-performed as-cast OSCs (refer to Table S5 for comparisons). To further elevate performance, we introduced 0.5% (by volume) 1,8-diiodooctane into the active layer and implemented a thermal annealing step at 80 °C for ten minutes. This optimization strategy improved the device’s efficiency to an even higher value of 19.23%, accompanied by a *V*_OC_ of 849 mV, a *J*_SC_ of 29.08 mA cm⁻^2^, and an FF of 77.83% (depicted in Fig. S6).

Finally, we evaluated the long-term stability and the universality of the dual-donor induced crystallinity modulation strategy. Device stability is crucial for solar cell performance [50, 51]. As shown in Fig. S14, after 800 h of indoor light exposure, the ternary device retains 82% of its initial PCE. Even at 60 °C, the ternary device exhibits greater stability than the binary device, indicating that the addition of PTzBI-dF enhances the device’s lifetime. We also investigated the universality of this strategy by applying it to several host systems (D18-Cl:L8-BO-x, D18-Cl:L8-BO, D18-Cl:BTP-eC9). The results, detailed in Fig. S15 and Table S17, show that the dual-donor approach positively impacts photovoltaic parameters, confirming its broad applicability.

## Conclusions

In summary, we demonstrated that PTzBI-dF, structurally similar to D18 and its derivatives, effectively modulated the active layer morphology of the ternary blend, resulting in a significantly enhanced performance exceeding 19%. The advanced crystalline structures induced by PTzBI-dF, along with its well-aligned energy levels and complementary absorption spectra, were critical to these performance improvements. Incorporating PTzBI-dF reduced current leakage, improved charge carrier mobilities, suppressed nongeminate charge recombination, and reduced energy losses. These findings confirm that introducing materials with structural similarity, but different aggregation features is a direct and effective approach to optimize the active layer. This provides valuable guidelines for designing the third component in efficient ternary OSCs.

## Supplementary Information

Below is the link to the electronic supplementary material.

Supplementary file1 (DOCX 2570 kb)
