# Supplementary material for: Dual-Donor-Induced Crystallinity Modulation Enables 19.23% Efficiency Organic Solar Cells
Source: Nanomicro Lett. 2024 Nov 27;17:72. doi: 10.1007/s40820-024-01576-1 (PMC11602937; doi:10.1007/s40820-024-01576-1)
Supplement: Supplementary file 1 — Supplementary file1 (DOCX 2570 kb) [file 40820_2024_1576_MOESM1_ESM.docx]

Supporting Information for

**Dual-Donor-Induced Crystallinity Modulation Enables 19.23% Efficiency Organic Solar Cells**

Anhai Liang^1^, Yuqing Sun^1^, Sein Chung^2^, Jiyeong Shin^2^, Kangbo Sun^3^, Chaofeng Zhu^1^, Jingjing Zhao^1^, Zhenmin Zhao^1^, Yufei Zhong^4^, Guangye Zhang^3^, Kilwon Cho^2^, Zhipeng Kan^1,5^ *

^1^ Center on Nanoenergy Research, Institute of Science and Technology for Carbon Peak & Neutrality, School of Physical Science & Technology, Guangxi University, Nanning 530004, P. R. China

^2^ Department of Chemical Engineering, Pohang University of Science and Technology, Pohang 37673, South Korea

^3^ College of New Materials and New Energies, Shenzhen Technology University, Shenzhen 518118, P. R. China

^4^ Zhejiang Engineering Research Center for Fabrication and Application of Advanced Photovoltaic Materials, School of Materials Science and Engineering, NingboTech University, Ningbo 315100, P. R. China

^5^ State Key Laboratory of Featured Metal Materials and Life-cycle Safety for Composite Structures, Nanning 530004, P. R. China

*Corresponding author. E-mail: [kanzhipeng@gxu.edu.cn](mailto:kanzhipeng@gxu.edu.cn) (Zhipeng Kan)

**S1 Ultraviolet photoelectron spectroscopy (UPS) measurements**

Ultraviolet photoelectron spectroscopy (UPS) measurements were performed in Kratos AXIS ULTRA DLD ultra-high vacuum photoelectron spectroscopy with base pressures greater than 2 × 10^-9^ Torr. The original film was obtained by spinning a solution containing different proportions of D18-Cl, PTzBI-dF and Y6 onto a 1 cm × 1 cm Ocoated glass substrate. After heating for 2 hours at 120 °C in a glove box filled with pure nitrogen, these substrates were immediately transferred to a photoelectron spectroscopy chamber for UPS measurements using an unfiltered He-discharge lamp (21.22 eV) as an excitation source. The secondary electron cutoff spectra of the samples were obtained at a negative bias voltage of 5.0 V relative to the electronic analyzer.

**S2 UV-Vis absorption**

The UV-VIS absorption spectra of different blends were recorded on the PerkinElmer LAMBDA 365 UV-VIS spectrophotometer.

**S3 Photoluminescence characterizations**

The photoluminescence of the films was measured by a FLS1000 equipped with an integrating hemisphere at an excitation wavelength of 500 nm and 630 nm from Edinburgh Instruments Co., Ltd.

**S4 SCLC Measurements**

The carrier mobility (hole and electron mobility) of the photoactive layer was measured by fitting the dark current of the hole/electron diode with the space charge limited current (SCLC) model. The scan started from -5 V to 5 V. The measurements of pure electronic devices and pure hole devices are prepared. Electronic device structure of ITO/ZnO/BHJ/ZnO/Ag and only hole of ITO/PEDOT: PSS/BHJ/MoO3/Ag. The single-carrier device was connected to a Source Measure Unit (Keithley, Model 236 SMU) which provides DC9 voltage to the electron-only devices. The J−V values of different DC voltages can bedetected and recorded through SMU. The J-V characteristics were further analyzed by the space-charge-limited-current (SCLC) method to extract zero-field carrier mobilities, where SCLC is described by:

$$J=\frac{9\varepsilon_{O}\varepsilon_{\gamma}\mu_{0}V^{2}}{{8L}^{3}}exp(0.89\beta\sqrt{\frac{V}{L}})$$

where J is the current density, L is the film thickness of the active layer, µ_0_ is the hole or electron mobility, ε_r_ is the relative dielectric constant of the transport medium, ε_0_ is the permittivity of free space (8.85 × 10^-12^ F m^-1^), V (= V_appl_ - V_bi_) is the internal voltage in the device, where V_appl_ is the applied voltage to the device and V_bi_ is the built-in voltage due to the relative work function difference of the two electrodes.

**S5 Carrier extraction by linearly increasing voltage (CELIV)**

According to the composite model of Mozer et al., the change of carrier density n(t) with time can be expressed as:

$$n\left( t \right)=\frac{n_{0}}{1+{(\frac{t}{\tau_{b}})}^{\gamma}}$$

Where n(t) is the charge density at time t, n_0_ is the initial charge density, 𝜏_𝑏_ is the recombination lifetime, and γ is the dispersion parameter. γ closes to 1, indicating a nondispersive bimolecular recombination at room temperature. The closer γ is to 1, the fewer traps there are in the system, and the slower the rate of bimolecular recombination. In the dispersive bimolecular recombination, the decay of carrier density is given by:

$$\beta\left( t \right)=﹣\frac{dn(t)/d(t)}{n^{2}(t)}$$

where n(t) is the carrier density and 𝛽(t) is dispersive bimolecular recombination rate at a delay time t. Substituting the first equation, the bimolecular recombination rate 𝛽(t) can also be expressed as:

$$\beta\left( t \right)=\left( \frac{1}{\tau_{b}} \right)\gamma{n_{0}}^{-1}{(t/\tau_{b})}^{\gamma-1}$$

The resulting 𝛽(t) can be calculated from the fitting parameters n_0_, 𝜏_𝑏_ and 𝛾 using equation above.

**S6 Atomic force microscopy (AFM)**

The glass substrate is exposed to ultraviolet ozone plasma for 15 minutes and spin coated directly in a nitrogen environment. The atomic force microscope is used for non-contact scanning, and the cantilever oscillates at a distance of 5 ~ 10 nm above the sample surface when detecting the sample surface. By continuously changing the scale from 500 μm to 5 nm, the surface morphology of the film was observed, and finally the AFM image with the size of 2 x 2 μm was captured.

**S7 Transmission electron microscopy (TEM)**

A layer of PEDOT: PSS is applied to the base, and then spin the film. The glass substrate was placed on the surface of deionized water, dissolved in water using the hydrophilicity of the PEDOT: PSS layer, then transferred to a 50-mesh copper grid on the substrate (China Electron Microscope), and corrected electron microscope (Titan ETEM G2 E-Twin) by spherical aberration observation at an accelerated operating voltage of 300 keV.

**S8 Contact angle measurements**

The contact angles of the neat film and the blended film were performed on the L2004A1 (Ossila England) contact Angle instrument. Then the surface free energy was calculated by Owens-Wendt method:

$$\varepsilon_{L}\times\left( 1+\cos\theta\right)=2\times{(\varepsilon_{L}^{d}\cdot\varepsilon_{SV}^{d})}^{1/2}+2\times{(\varepsilon_{L}^{p}\cdot\varepsilon_{SV}^{p})}^{1/2}$$

Where *ε_L_* and *ε_S_* are the surface free energies of the sample film in different liquids (deionized water and formamide), θ is the contact Angle of the sample, the average contact Angle and surface energy parameters obtained through video dynamic analysis are shown in Table S6 and Table S7. Then the Flory Huggins interaction parameter χ_donor-acceptor_ of the blend is calculated to show the compatibility of the blend films, which can be obtained from the following formula:

$$\chi_{donor-acceptor}=K{(\gamma_{donor}^{1/2}-\gamma_{acceptor}^{1/2})}^{2}$$

where *γ* is the surface energy of the material, K is the proportionality constant.

**S9 Grazing incidence wide-angle x-ray scattering**

Swept grazing incidence wide-angle X-ray scattering (GIWAXS), beamlines 3C SAXS-I and 9A U-SAXS were measured at the Pohang Light Source in South Korea. After careful selection, the incidence Angle is 0.12° to ensure that the X-ray completely penetrates the film. The e film is prepared according to the method described in the section on Device Fabrication.

**Supplementary Figures and Tables**

**
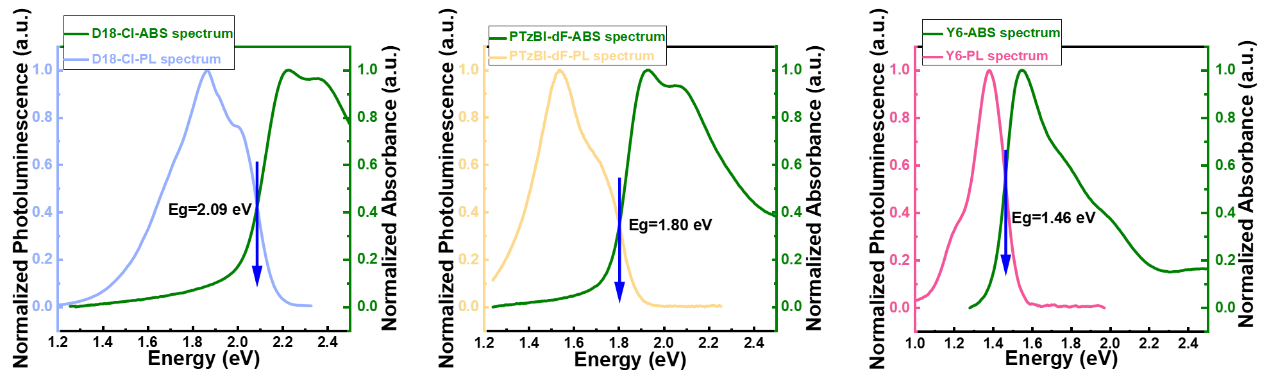
**

**Fig. S1** Normalized absorbance and photoluminescence spectra of the D18-Cl, PTzBI-dF and Y6

**
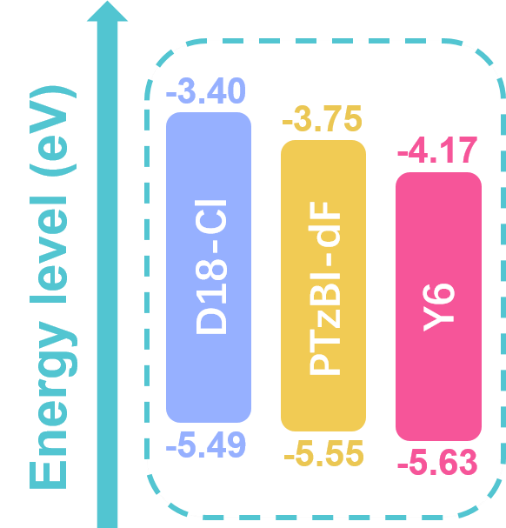
**

**Fig. S2** Schematic energy level diagram for D18-Cl、PTzBI-dF and Y6

**
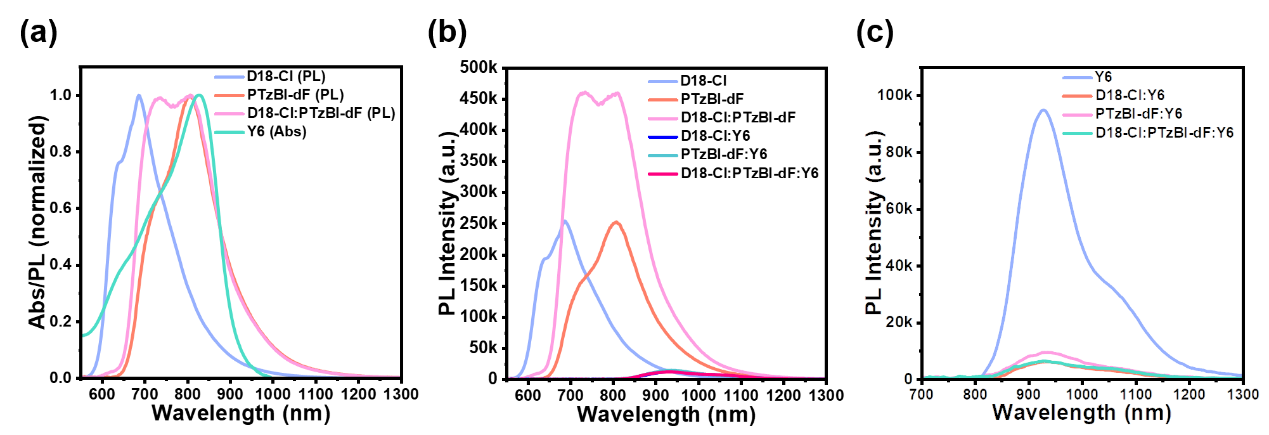
**

**Fig. S3** (**a**) Normalized the photoluminescence spectrum of D18-Cl、PTzBI-dF and D18-Cl:PTzBI-dF films and UV−vis absorption spectra of Y6 film. PL fluorescence emission spectrum films of D18-Cl, PTzBI-dF and Y6 neat and blended films: (**b**) donor excitation wavelength of 500nm and (**c**) acceptor excitation wavelength of 630nm


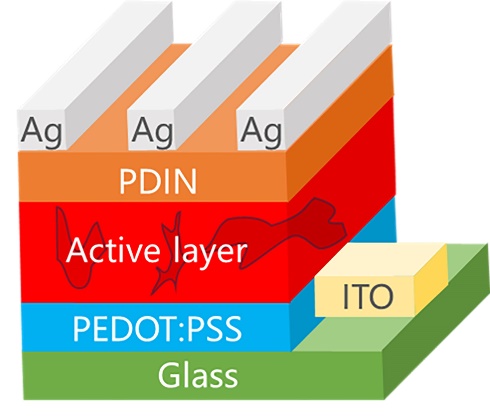


**Fig. S4** Structure diagram of OSCs conventional devices

**
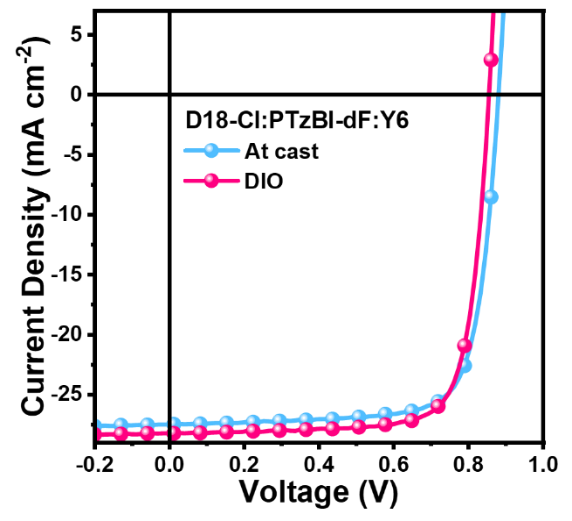
**

**Fig. S5** *J*-*V* of devices with different conditions


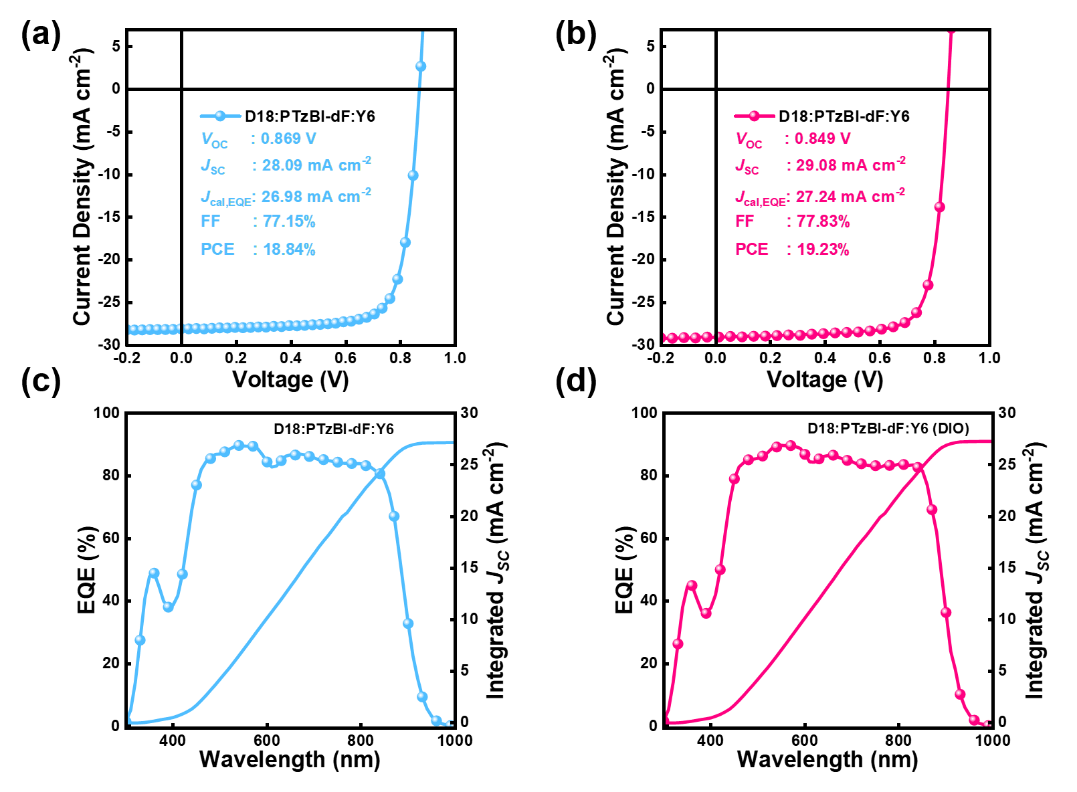


**Fig. S6** The *J*-*V* traces of D18: PTzBI-dF: Y6 ternary OSCs: (**a**) the at-cast; (**b**) DIO. (**c-d**) the external quantum efficiency (EQE) spectra of D18: PTzBI-dF: Y6 and D18: PTzBI-dF: Y6 (DIO)


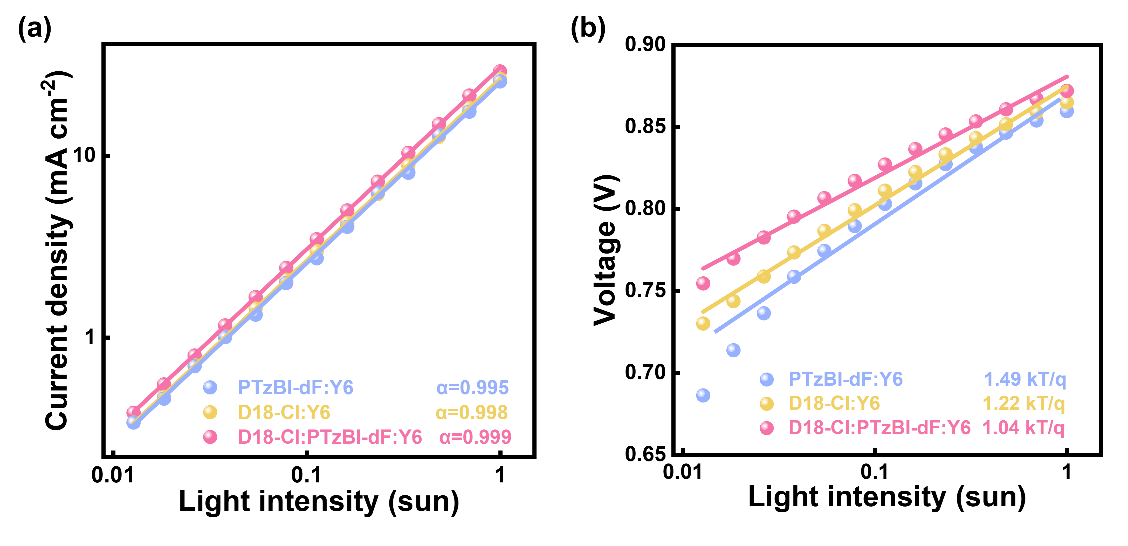


**Fig. S7** (**a**) *J_SC_* and (**b**) *V_OC_* dependence on the light intensity

**
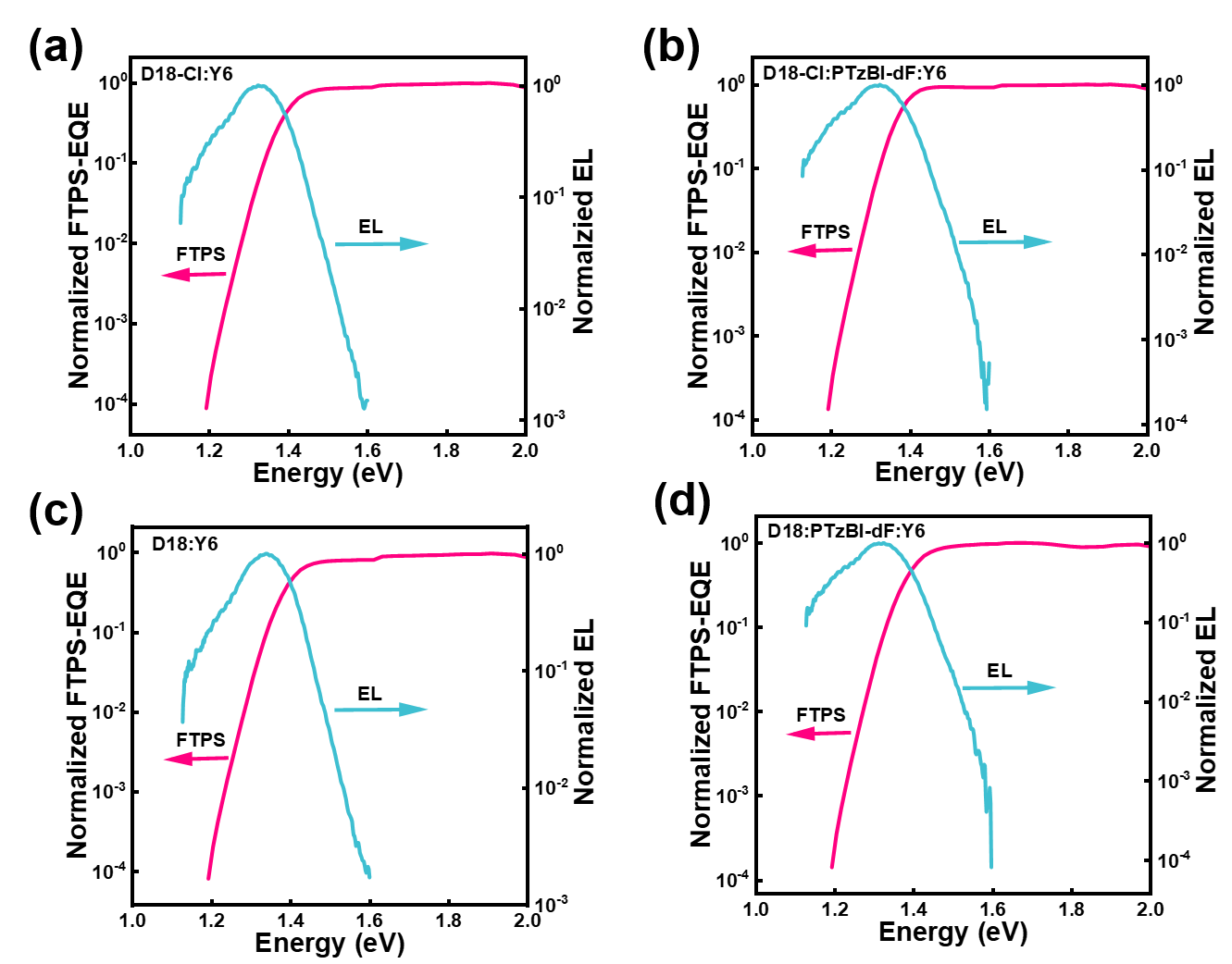
**

**Fig. S8** (**a-d**) Normalized EQE and EL spectra of binary and ternary devices


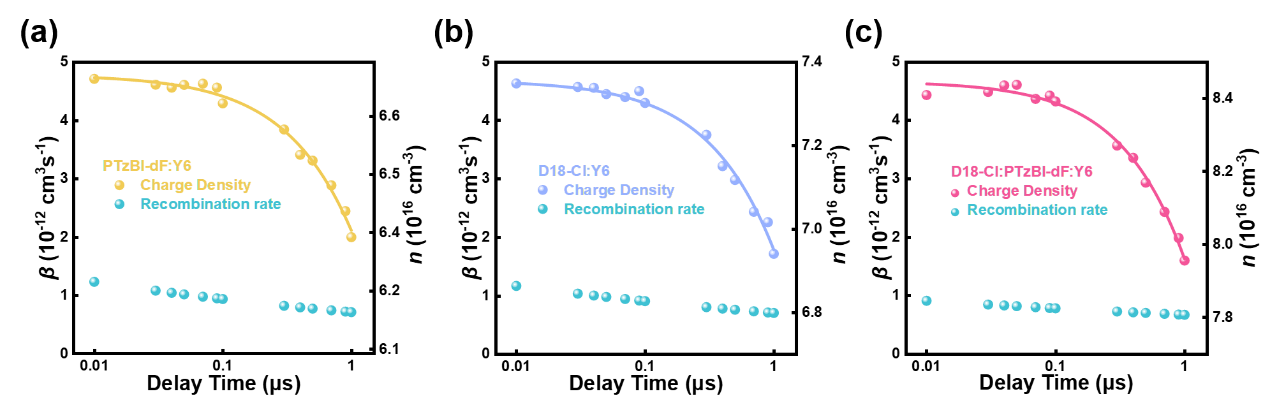


**Fig. S9** Charge extraction and recombination with various delay times of the devices: (**a**) PTzBI-dF: Y6, (**b**) D18-Cl: Y6, (**c**) D18-Cl: PTzBI-dF: Y6
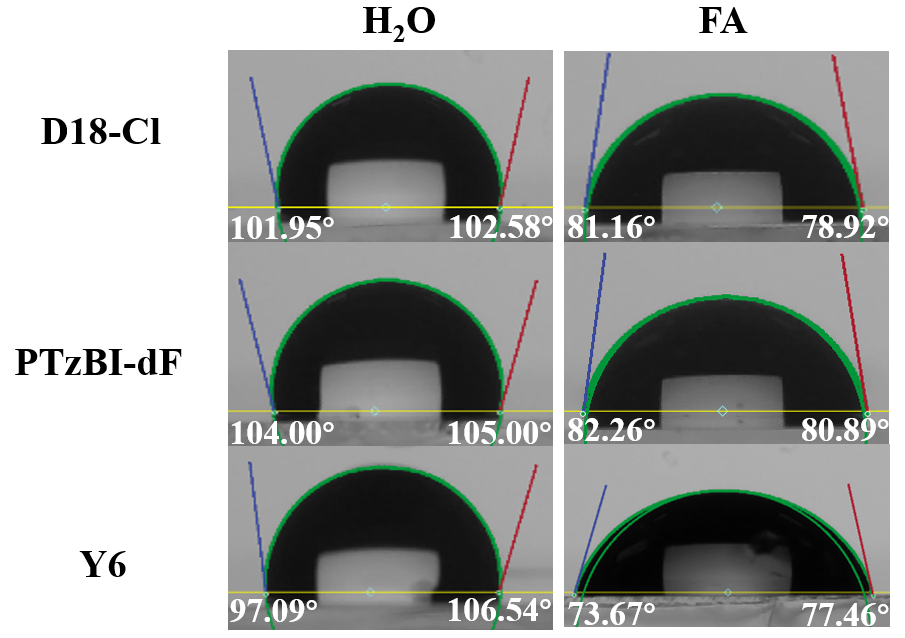


**Fig. S10** The contact angles of the neat films in deionized water (H_2_O) and formamide (FA)


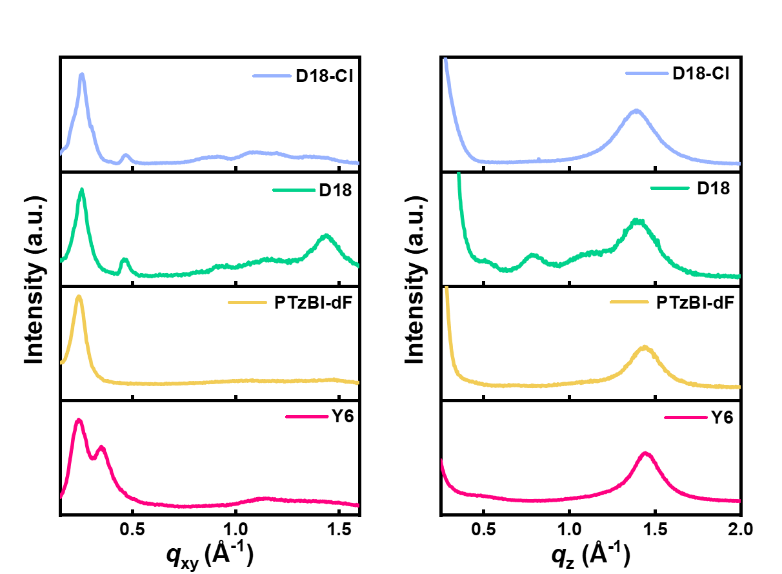


**Fig. S11** The in-plane (IP) and out-of-plane (OOP) line cuts of the neat D18-Cl, D18, PTzBI-dF, and Y6

**
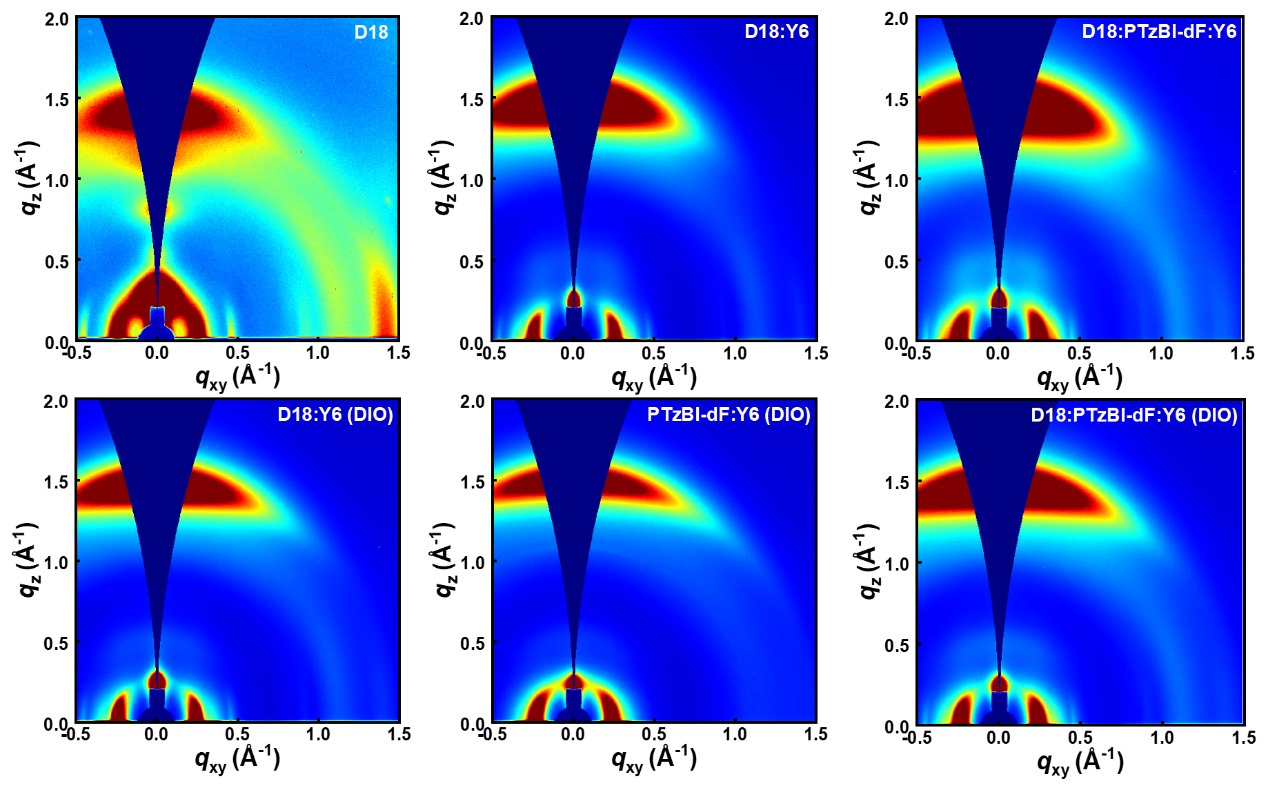
**

**Fig. S12** 2D GIWAXS patterns for D18: PTzBI-dF: Y6 ternary devices

**
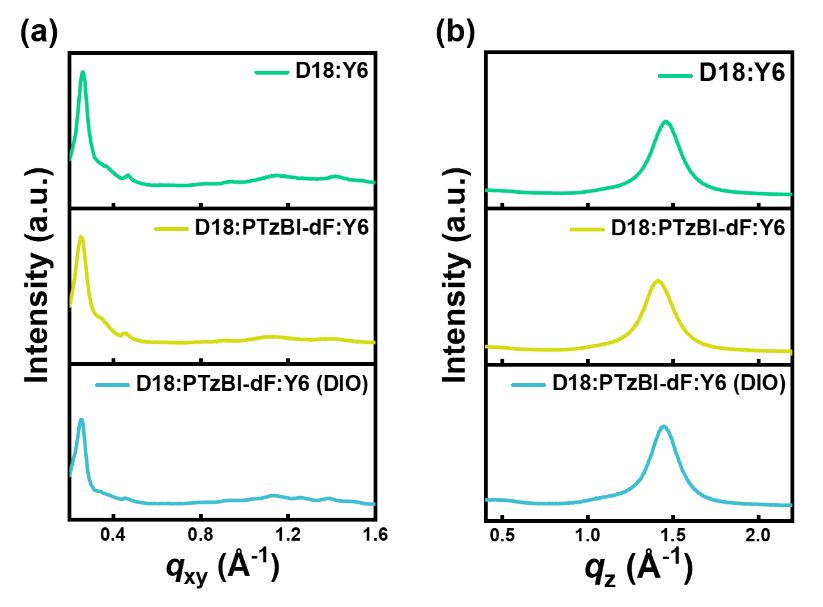
**

**Fig. S13** The in-plane and out-of-plane line cuts of the D18: PTzBI-dF: Y6 ternary devices

**
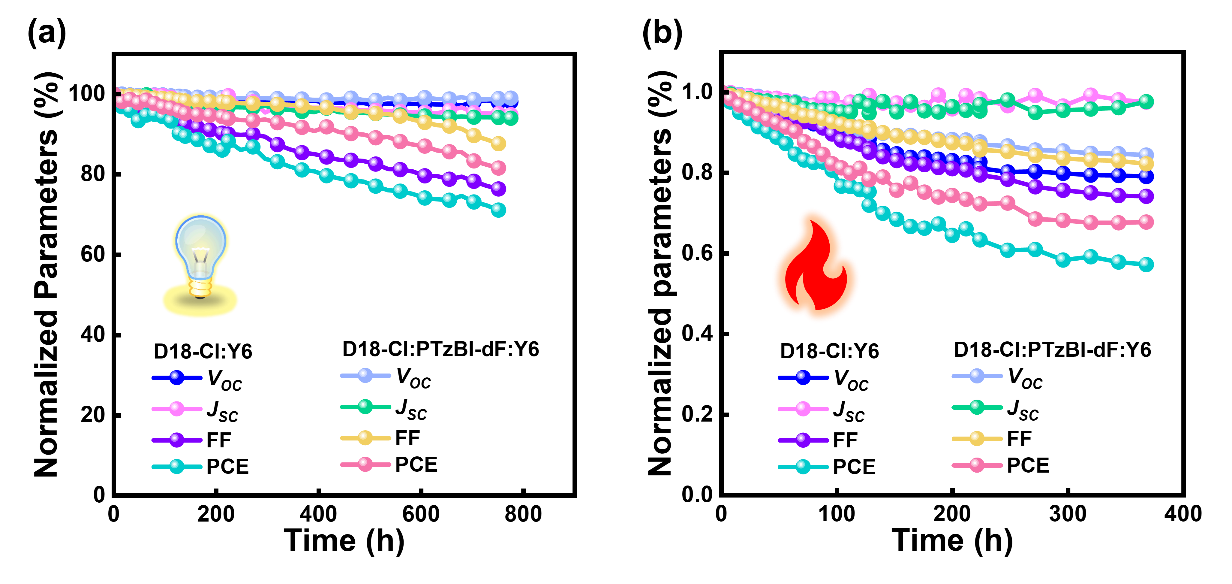
**

**Fig. S14** (**a**) Stability of the device under indoor light exposure. (**b**) Thermal stability of the device under conditions of 60 °C

**
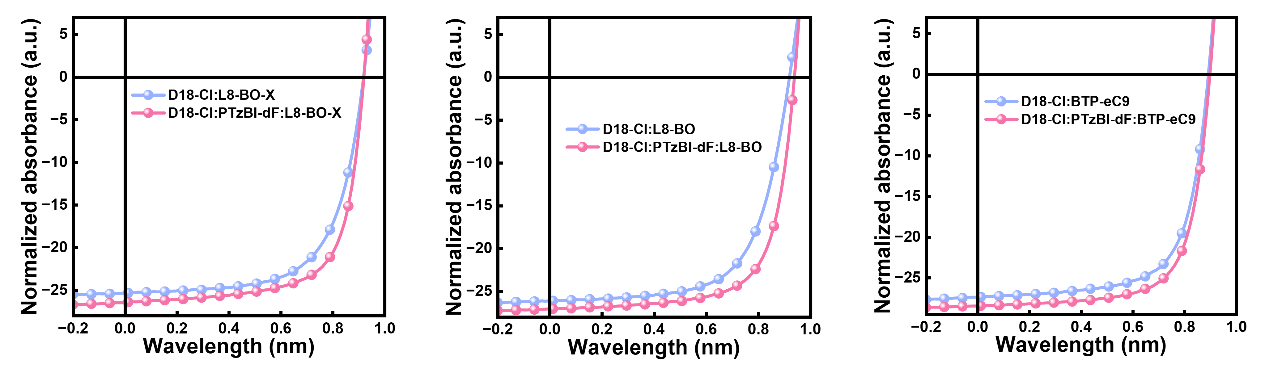
**

**Fig. S15** The optimal *J-V* curves of binary and ternary solar cells

**Table S1** The energy levels of the materials were studied by ultraviolet photoelectron spectroscopy

| Material | Highest occupied molecular orbitals (HOMO) | Optical band gaps  energy(eV) | Lowest unoccupied molecular orbitals (LUMO) |
| --- | --- | --- | --- |
| D18-Cl | -5.49 | 2.09 | -3.40 |
| PTzBI-dF | -5.55 | 1.80 | -3.75 |
| Y6 | -5.63 | 1.46 | -4.17 |

**Table S2** Photovoltaic parameters of the ternary OSCs with varied D18-Cl: PTzBI-dF ratios under the illumination of AM 1.5G, 100 mW cm^-2^

| Photoactive layer | *V*_OC_ (mV) | *J*_SC_ (mA cm^-2^) | FF (%) | PCE (%) |
| --- | --- | --- | --- | --- |
| D18-Cl: PTzBI-dF: Y6  (0.9:0.1:1.6) | 875  (877±2) | 27.40  (27.18±0.15) | 76.44  (76.40±0.14) | 18.33  (18.22±0.06) |
| D18-Cl: PTzBI-dF: Y6  (0.8:0.2:1.6) | 882  (880±1) | 27.50  (27.40±0.12) | 76.66  (76.55±0.10) | 18.60  (18.45±0.06) |
| D18-Cl: PTzBI-dF: Y6  (0.7:0.3:1.6) | 869  (871±1) | 27.42  (27.18±0.18) | 76.46  (76.43±0.09) | 18.22  (18.09±0.11) |
| D18-Cl: PTzBI-dF: Y6  (0.6:0.4:1.6) | 865  (868±2) | 28.61  (27.88±0.37) | 71.98  (70.89±0.45) | 17.81  (17.43±0.13) |
| D18-Cl: PTzBI-dF: Y6  (0.5:0.5:1.6) | 866  (862±3) | 27.84  (27.01±0.31) | 68.03  (68.30±0.55) | 16.40  (15.96±0.16) |

**Table S3** The *J*_cal, EQE_ from EQE spectra and the *J*_SC_ measured under the illumination of AM 1.5G, 100 mW cm^-2^

| Photoactive layer | *J*_SC_ [mA cm^-2^] | *J*_cal, EQE_[mA cm^-2^] |
| --- | --- | --- |
| PTzBI-dF:Y6 | 25.63 | 25.41 |
| D18-Cl:Y6 | 26.90 | 26.19 |
| D18-Cl: PTzBI-dF: Y6  (0.8:0.2:1.6) | 27.50 | 27.00 |

**Table S4** Summary of photovoltaic parameters of the D18-Cl: PTzBI-dF: Y6 devices processed with different conditions

| Conditions | *V*_OC_ (mV) | *J*_SC_ (mA cm^-2^) | FF (%) | PCE (%) |
| --- | --- | --- | --- | --- |
| Control | 882  (880±1) | 27.50  (27.40±0.12) | 76.66  (76.55±0.10) | 18.60  (18.45±0.06) |
| DIO | 855  (856±1) | 28.23  (27.98±0.18) | 77.52  (77.48±0.18) | 18.71  (18.52±0.11) |

**Table S5** Detailed device parameters of reported representative as-cast OSCs

| Photoactive layer | *V*_OC_  (mV) | *J*_SC_  (mA cm^-2^) | FF  (%) | PCE (%) | Year |
| --- | --- | --- | --- | --- | --- |
| PBTIBDTT:ITIC-F | 921 | 16.4 | 74.1 | 11.19 | 2018[1] |
| PTB7-Th:FOIC | 743 | 24.0 | 67.1 | 12.00 | 2018[2] |
| PBDB-T/m-INPOIC/P71BM | 857 | 22.8 | 71.6 | 14.00 | 2019[3] |
| PM6:IDIC:TOBDT | 920 | 21.4 | 71.0 | 14.00 | 2020[4] |
| D18:Y6Se | 839 | 27.98 | 75.3 | 17.70 | 2020[5] |
| PM6:BTP-ClBr1:BTP-2O-4Cl-C12 | 896 | 24.86 | 74.8 | 16.68 | 2021[6] |
| PM6:Y6:IT-M | 855 | 26.91 | 68.7 | 15.80 | 2021[7] |
| PBDB-T:L5 | 860 | 24.67 | 71.36 | 15.20 | 2022[8] |
| PM6:Y6:L5 | 870 | 27.81 | 71.00 | 17.14 | 2022[8] |
| PM6:Y6 | 857 | 25.74 | 75.11 | 16.57 | 2022[9] |
| PM6:BTP-4Cl:PC71BM | 865 | 26.05 | 77.00 | 17.36 | 2022[10] |
| PM6:Y6-eC6-BO | 846 | 26.44 | 77.42 | 17.33 | 2023[11] |
| PBDB-T:BOR-C4Ph | 827 | 22.67 | 70.03 | 13.12 | 2023[12] |
| D18:L8-BO:PY-TPT | 926 | 25.67 | 78.27 | 18.60 | 2024[13] |
| D18-Cl:PTzBI-dF:Y6 | 882 | 27.50 | 76.66 | 18.60 | This work |
| D18:PTzBI-dF:Y6 | 869 | 28.09 | 77.15 | 18.84 | This work |

**Table S6** Summary of D18 donor-based OSCs

| Photoactive layer | *V_OC_*  (mV) | *J_SC_*  (mA cm^-2^) | FF  (%) | PCE (%) | Ref. |
| --- | --- | --- | --- | --- | --- |
| D18/Y6 | 859 | 27.24 | 76.67 | 17.94 | [14] |
| D18:Y6 | 859 | 27.70 | 76.60 | 18.22 | [15] |
| D18:Y6:BTP-H2-γ | 871 | 27.12 | 78.38 | 18.51 | [16] |
| D18:Y6:BTP-H2-δ | 855 | 27.01 | 78.07 | 18.03 | [16] |
| D18-Cl-B_M_:N3:PC_61_BM | 836 | 28.50 | 78.7 | 18.74 | [17] |
| D18-B_M_:N3:PC_61_BM | 825 | 28.50 | 79.0 | 18.53 | [17] |
| D18:PTzBI-dF:Y6 | 849 | 29.08 | 77.83 | 19.23 | This work |

**Table S7** Summarized *μ*_e_ and *μ*_h_ for PTzBI-dF:Y6、D18-Cl:Y6 and D18-Cl:PTzBI-dF:Y6

| Photoactive layer | *µ*_e_ (cm^2^ V^-1^ s^-1^) | *µ*_h_ (cm^2^ V^-1^ s^-1^) | *µ*_e_/*µ*_h_ |
| --- | --- | --- | --- |
| PTzBI-dF:Y6 | 5.31×10^-4^ | 8.62×10^-4^ | 0.62 |
| D18-Cl:Y6 | 9.18×10^-4^ | 7.89×10^-4^ | 1.16 |
| D18-Cl: PTzBI-dF: Y6  (0.8:0.2:1.6) | 11.6×10^-4^ | 11.0×10^-4^ | 1.05 |

**Table S8** Summarized dispersive bimolecular recombination fitting parameters for D18-Cl:Y6、PTzBI-dF:Y6 and D18-Cl: PTzBI-dF: Y6

| Photoactive layer | n_0_ (cm^-3^ ) | γ | τ_b_ (10^-5^ µs) | β(10^-12^ cm^3^ s^-1^) |
| --- | --- | --- | --- | --- |
| PTzBI-dF:Y6 | 6.72×10^16^ | 0.88 | 2.72 | 1.23 |
| D18-Cl:Y6 | 7.36×10^16^ | 0.89 | 2.43 | 1.17 |
| D18-Cl:PTzBI-dF:Y6 | 8.45×10^16^ | 0.93 | 2.00 | 0.91 |

**Table S9** Summarized Contact Angles and Surface Free Energy Parameters of the materials

| Films | Contact angle (dge) | | Surface free energy,  γ(mJ m^-2^) | χ_donor−acceptor_^b^  (×10^-2^K) |
| --- | --- | --- | --- | --- |
|  | H_2_O^a^ | FA |  |  |
| D18-Cl | 102.27 | 80.04 | 24.25 | 29.46 |
| PTzBI-dF | 104.50 | 81.58 | 24.13 | 30.80 |
| Y6 | 101.81 | 75.57 | 29.89 | -- |

^a^Deionized water; ^b^Estimates for Flory−Huggins interaction parameter (χ_donor−acceptor_).

**Table S10** Summarizes the contact Angle and surface free energy parameters of donors of different proportions

| Films  (D18-Cl:PTzBI-dF) | Contact angle (dge) | | Surface free energy,  γ(mJ m^-2^) | χ_donor−acceptor_^b^  (×10^-2^K) |
| --- | --- | --- | --- | --- |
|  | **H_2_O^a^** | **FA** |  |  |
| 0.9:0.1 | 104.68 | 76.93 | 30.97 | 9.58 |
| 0.8:0.2 | 104.47 | 79.16 | 27.41 | 5.37 |
| 0.7:0.3 | 100.67 | 77.55 | 26.11 | 12.77 |
| 0.6:0.4 | 103.84 | 80.74 | 24.66 | 25.13 |
| 0.4:0.6 | 106.05 | 81.28 | 25.96 | 13.84 |
| 0.2:0.8 | 105.26 | 81.30 | 25.18 | 20.18 |

^a^ Deionized water; ^b^ Estimates for Flory−Huggins interaction parameter (χ_donor−acceptor_).

**Table S11** The peak position and CCL in IP directions of neat D18-Cl and PTzBI-dF

| Component | Peak position  (Å^-1^) | *d*-spacing  (Å) | FWHM  (Å^-1^) | Crystalline Coherence Length^a^  (Å) |
| --- | --- | --- | --- | --- |
| D18-Cl | 0.258 | 24.35 | 0.033 | 171.36 |
| D18 | 0.250 | 25.13 | 0.041 | 153.25 |
| PTzBI-dF | 0.244 | 25.75 | 0.060 | 90.25 |
| Y6 | 0.238 | 26.40 | 0.074 | 76.42 |

^a^ Using Scherrer equation to calculate coherence length (CCL) Peak CCL = 2πk/Δ*q*, where k is a dimensionless shape factor (where k = 0.9) and Δ*q* is the full half-peak width (FWHM) of a given peak.

**Table S12** The peak position and CCL in OOP directions of neat D18-Cl and PTzBI-dF

| Component | Peak position  (Å^-1^) | *d*-spacing  (Å) | FWHM  (Å^-1^) | Crystalline Coherence Length^a^  (Å) |
| --- | --- | --- | --- | --- |
| D18-Cl | 1.392 | 4.51 | 0.183 | 30.90 |
| D18 | 1.411 | 4.45 | 0.180 | 31.42 |
| PTzBI-dF | 1.443 | 4.35 | 0.195 | 28.99 |
| Y6 | 1.452 | 4.33 | 0.159 | 35.56 |

^a^ Using Scherrer equation to calculate coherence length (CCL) Peak CCL = 2πk/Δ*q*, where k is a dimensionless shape factor (where k = 0.9) and Δ*q* is the full half-peak width (FWHM) of a given peak.

**Table S13** The peak position and CCL in IP directions of D18-Cl:Y6、PTzBI-dF:Y6 and D18-Cl: PTzBI-dF: Y6

| Component | Peak | Peak position(Å^-1^) | *d*-spacing  (Å) | FWHM  (Å^-1^) | Crystalline Coherence Length^a^(Å) |
| --- | --- | --- | --- | --- | --- |
| D18-Cl:Y6 | IP(010) | 0.291 | 21.59 | 0.173 | 32.69 |
|  | IP(100) | 0.254 | 24.74 | 0.074 | 76.42 |
| PTzBI-dF:Y6 | IP(010) | 0.274 | 22.93 | 0.190 | 29.76 |
|  | IP(100) | 0.244 | 25.75 | 0.071 | 79.65 |
| D18-Cl:PTzBI-dF:Y6 | IP(010) | 0.297 | 21.16 | 0.177 | 31.95 |
|  | IP(100) | 0.250 | 25.13 | 0.077 | 73.44 |

**Table S14** The peak position and CCL in OOP directions of D18-Cl:Y6、PTzBI-dF:Y6 and D18-Cl: PTzBI-dF: Y6

| Component | Peak | Peak position  (Å^-1^) | *d*-spacing  (Å) | FWHM  (Å^-1^) | Crystalline Coherence Length^a^(Å) |
| --- | --- | --- | --- | --- | --- |
| D18-Cl:Y6 | D18-Cl | 1.459 | 4.31 | 0.177 | 31.94 |
|  | Y6 | 1.430 | 4.39 | 0.418 | 13.53 |
| PTzBI-dF:Y6 | PTzBI-dF | 1.461 | 4.30 | 0.179 | 31.59 |
|  | Y6 | 1.404 | 4.48 | 0.544 | 10.39 |
| D18-Cl:PTzBI-dF:Y6 | D18-Cl:PTzBI-dF | 1.458 | 4.31 | 0.173 | 32.69 |
|  | Y6 | 1.430 | 4.39 | 0.408 | 13.86 |

**Table S15** The peak position and CCL in IP directions of D18:Y6、D18:PTzBI-dF: Y6 and D18: PTzBI-dF:Y6 (DIO)

| Component |  | Peak | Peak position(Å^-1^) | *d*-spacing  (Å) | FWHM  (Å^-1^) | Crystalline Coherence Length^a^(Å) |
| --- | --- | --- | --- | --- | --- | --- |
| D18:Y6 |  | IP(010) | 0.259 | 24.26 | 0.154 | 36.72 |
|  |  | IP(100) | 0.259 | 24.26 | 0.045 | 125.66 |
| D18:PTzBI-dF: Y6 |  | IP(010) | 0.273 | 23.02 | 0.178 | 31.77 |
|  |  | IP(100) | 0.250 | 25.13 | 0.059 | 95.84 |
| D18: PTzBI-dF: Y6  (DIO) |  | IP(010) | 0.278 | 22.60 | 0.262 | 21.58 |
|  |  | IP(100) | 0.249 | 25.23 | 0.060 | 94.25 |

**Table S16** The peak position and CCL in OOP directions of D18:Y6、D18:PTzBI-dF: Y6 and D18: PTzBI-dF: Y6 (DIO)

| Component | Peak | Peak position  (Å^-1^) | *d*-spacing  (Å) | FWHM  (Å^-1^) | Crystalline Coherence Length^a^(Å) |
| --- | --- | --- | --- | --- | --- |
| D18:Y6 | D18 | 1.462 | 4.30 | 0.166 | 34.06 |
|  | Y6 | 1.439 | 4.37 | 0.437 | 13.94 |
| D18:PTzBI-dF:Y6 | D18:PTzBI-dF | 1.418 | 4.43 | 0.165 | 34.27 |
|  | Y6 | 1.402 | 4.48 | 0.380 | 14.88 |
| D18: PTzBI-dF: Y6  (DIO) | D18:PTzBI-dF | 1.451 | 4.33 | 0.161 | 35.12 |
|  | Y6 | 1.417 | 4.43 | 0.381 | 14.84 |

**Table S17** Photovoltaic parameters of the optimum binary and ternary blend films under AM 1.5G and 100 mW cm^-2^ illumination

| Photoactive layer | *V*_OC_ [mV] | *J*_SC_ [mA cm^-2^] | FF [%] | PCE [%] |
| --- | --- | --- | --- | --- |
| D18-Cl:L8-BO-X | 919  (915±2) | 25.33  (25.08±0.23) | 65.24  (64.75±0.20) | 15.19  (15.01±0.13) |
| PTzBI-dF: L8-BO-X | 892  (893±3) | 25.17  (24.62±0.50) | 53.54  (53.04±0.83) | 12.02  (11.59±0.22) |
| D18-Cl:PTzBI-dF: L8-BO-X | 921  (923±2) | 26.45  (25.92±0.23) | 69.47  (69.16±0.20) | 16.92  (16.55±0.16) |
| D18-Cl:L8-BO | 920  (918±2) | 26.12  (25.89±0.19) | 65.25  (64.61±0.32) | 15.68  (15.48±0.11) |
| PTzBI-dF: L8-BO | 916  (910±3) | 24.67  (23.77±0.58) | 51.37  (49.95±0.86) | 11.60  (10.82±0.38) |
| D18-Cl:PTzBI-dF: L8-BO | 939  (936±2) | 27.07  (26.82±0.17) | 70.21  (69.73±0.26) | 17.84  (17.41±0.14) |
| D18-Cl:BTP-eC9 | 893  (892±1) | 27.38  (26.79±0.26) | 68.48  (67.87±0.27) | 16.75  (16.47±0.12) |
| PTzBI-dF: BTP-eC9 | 880  (878±2) | 26.89  (26.08±0.41) | 65.97  (65.00±0.70) | 15.60  (15.08±0.23) |
| D18-Cl:PTzBI-dF: BTP-eC9 | 898  (893±2) | 28.47  (27.33±0.56) | 70.62  (70.13±0.11) | 18.06  (17.62±0.31) |

**Supplementary References**

1. T. Zhang, G. Zeng, F. Ye, X. Zhao, X. Yang, Efficient non-fullerene organic photovoltaic modules incorporating as-cast and thickness-insensitive photoactive layers. Adv. Energy Mater. **8**, 1801387 (2018). <https://doi.org/10.1002/aenm.201801387>
2. T. Li, S. Dai, Z. Ke, L. Yang, J. Wang, et al., Fused tris(thienothiophene)-based electron acceptor with strong near-infrared absorption for high-performance as-cast solar cells. Adv. Mater. **30**, 1705969 (2018).  <https://doi.org/10.1002/adma.201705969>
3. H. Feng, X. Song, Z. Zhang, R. Geng, J. Yu, et al., Molecular orientation unified nonfullerene acceptor enabling 14% efficiency as-cast organic solar cells. Adv. Funct. Mater. **29**, 1903269 (2019). <https://doi.org/10.1002/adfm.201903269>
4. X. Chen, B. Kan, Y. Kan, M. Zhang, S. B. Jo, et al., As-cast ternary organic solar cells based on an asymmetric side-chains featured acceptor with reduced voltage loss and 14.0% efficiency. Adv. Funct. Mater. **30**, 1909535 (2020). <https://doi.org/10.1002/adfm.201909535>
5. Z. Zhang, Y. Li, G. Cai, Y. Zhang, X. Lu, et al., Selenium heterocyclic electron acceptor with small urbach energy for as-cast high-performance organic solar cells. J. Am. Chem. Soc. **142**, 18741-18745 (2020). <https://doi.org/10.1021/jacs.0c08557>
6. R. Ma, Y. Tao, Y. Chen, T. Liu, Z. Luo, et al., Achieving 16.68% efficiency ternary as-cast organic solar cells. Sci. China Chem. **64**, 581 (2021). https://doi.org/10.1007/s11426-020-9912-0
7. Y. Zhao, Y. Liu, X. Liu, X. Kang, L. Yu, et al., Aminonaphthalimide-based molecular cathode interlayers for as-cast organic solar cells. ChemSusChem. **14**, 4783-4792 (2021). <https://doi.org/10.1002/cssc.202101383>
8. D. Li, H. Lu, Y.-N. Chen, X. Ma, H. Zhang, et al., High-efficiency as-cast organic solar cells based on an asymmetric acceptor. Chem. Mater. **34**, 8840-8848 (2022). <https://doi.org/10.1021/acs.chemmater.2c02146>
9. K. Yu, W. Song, J. Ge, K. Zheng, L. Xie, et al., 18.01% Efficiency organic solar cell and 2.53% light utilization efficiency semitransparent organic solar cell enabled by optimizing PM6:Y6 active layer morphology. Sci. China Chem. **65**, 1615 (2022). https://doi.org/10.1007/s11426-022-1270-5
10. R. Ma, T. Yang, Y. Xiao, T. Liu, G. Zhang, et al., Air-processed efficient organic solar cells from aromatic hydrocarbon solvent without solvent additive or post-treatment: insights into solvent effect on morphology. Energy Environ. Mater. **5**, 977 (2022).  <https://doi.org/10.1002/eem2.12226>
11. X. Xu, Y. Qi, X. Luo, X. Xia, X. Lu, et al., Alkyl side chain engineering enables high performance as-cast organic solar cells of over 17% efficiency. Fundamental Research **3**, 611-617 (2023). <https://doi.org/10.1016/j.fmre.2022.01.025>
12. J. Wang, Q. Luan, P. Wang, C. Han, F. Bi, et al., Easily available high-performance organic solar cells by regulating phenylalkyl side groups of non-fused ring electron acceptors. Adv. Funct. Mater. 33, 2301575 (2023). <https://doi.org/10.1002/adfm.202301575>
13. Y. Wei, X. Zhou, Y. Cai, Y. Li, S. Wang, et al., High performance as-cast organic solar cells enabled by a refined double-fibril network morphology and improved dielectric constant of active layer. Adv. Mater. 2403294 (2024). <https://doi.org/10.1002/adma.202403294>
14. S. Liu, Y. Zhou, Z. Liang, B. Zhao, W. Wang, et al., High-performance pseudo-bilayer organic solar cells enabled by sequential deposition of D18/Y6 chloroform solution. ACS Appl. Energy Mater. **6**, 5047 (2023). <https://doi.org/10.1021/acsaem.3c00694>
15. Q. Liu, Y. Jiang, K. Jin, J. Qin, J. Xu, et al., 18% Efficiency organic solar cells. Science Bulletin **65**, 272-275 (2020). <https://doi.org/10.1016/j.scib.2020.01.001>
16. Y. Zhu, X. Shen, H. Lai, M. Pu, Y. Zhu, et al., Leveraging isomeric effect of third components in D18:Y6 system to 18.51% efficiency. Nano Energy **118**, 108991 (2023). <https://doi.org/10.1016/j.nanoen.2023.108991>
17. X. Meng, K. Jin, Z. Xiao, and L. Ding, Side chain engineering on D18 polymers yields 18.74% power conversion efficiency. J. Semicond. **42**, 100501 (2021). http://doi.org/10.1088/1674-4926/42/10/100501
